# Red Blood Cell Distribution Width Is Associated With Adverse Kidney Outcomes in Patients With Chronic Kidney Disease

**DOI:** 10.3389/fmed.2022.877220

**Published:** 2022-06-09

**Authors:** Xinwei Deng, Bixia Gao, Fang Wang, Ming-hui Zhao, Jinwei Wang, Luxia Zhang

**Affiliations:** ^1^Renal Division, Department of Medicine, Peking University First Hospital, Beijing, China; ^2^Institute of Nephrology, Peking University, Beijing, China; ^3^Research Units of Diagnosis and Treatment of Immune-Mediated Kidney Diseases, Chinese Academy of Medical Sciences, Beijing, China; ^4^Key Laboratory of Renal Disease, Ministry of Health of China, Beijing, China; ^5^Key Laboratory of Chronic Kidney Disease Prevention and Treatment, Ministry of Education of China, Beijing, China; ^6^Peking-Tsinghua Center for Life Sciences, Academy for Advanced Interdisciplinary Studies, Peking University, Beijing, China; ^7^National Institute of Health Data Science, Peking University, Beijing, China

**Keywords:** chronic kidney disease (CKD), rapid CKD progression, kidney outcomes, red blood cell distribution width, estimated glomerular filtration rate (eGFR) slope

## Abstract

**Background:**

Chronic kidney disease (CKD) is a global public health issue. Red blood cell distribution width (RDW) is a recently recognized potential inflammatory marker, which mirrors the variability in erythrocyte volume. Studies indicate that elevated RDW is associated with increased risk of mortality in CKD patients, while evidence regarding the impact of RDW on kidney outcome is limited.

**Methods:**

Altogether 523 patients with CKD stage 1–4 from a single center were enrolled. We identified the cutoff point for RDW level using maximally selected log-rank statistics. The time-averaged estimated glomerular filtration rate (eGFR) slope was determined using linear mixed effects models. Rapid CKD progression was defined by an eGFR decline >5 ml/min/1.73 m^2^/year. The composite endpoints were defined as doubling of serum creatinine, a 30% decline in initial eGFR or incidence of eGFR < 15 ml/min/1.73 m^2^, whichever occurred first. Multivariable logistic regression or Cox proportional hazards regression was performed, as appropriate.

**Results:**

During a median follow-up of 26 [interquartile range (IQR): 12, 36] months, 65 (12.43%) patients suffered a rapid CKD progression and 172 (32.89%) composite kidney events occurred at a rate of 32.3/100 patient-years in the high RDW group, compared with 14.7/100 patient-years of the remainder. The annual eGFR change was clearly steeper in high RDW group {−3.48 [95% confidence interval (CI): −4.84, −2.12] ml/min/1.73 m^2^/year vs. −1.86 [95% CI: −2.27, −1.45] ml/min/1.73 m^2^/year among those with RDW of >14.5% and ≤14.5%, respectively, *P* for between-group difference <0.05}. So was the risk of rapid renal function loss (odds ratio = 6.79, 95% CI: 3.08–14.97) and composite kidney outcomes (hazards ratio = 1.51, 95% CI: 1.02–2.23). The significant association remained consistent in the sensitivity analysis.

**Conclusion:**

Increased RDW value is independently associated with accelerated CKD deterioration. Findings of this study suggest RDW be a potential indicator for risk of CKD progression.

## Introduction

Chronic kidney disease (CKD) is a growing public health issue, affecting approximately 8–16% of global population, posing a significant public burden both socially and economically (1, 2). The co-occurrence of chronic malnutrition and inflammation is considered as a common pathophysiologic status in CKD progression, triggering deterioration of renal function and poor prognosis (3, 4). Measurable parameters in blood which could provide early detection of these specific conditions are of utmost importance.

Red blood cell distribution width (RDW) is a common coefficient of heterogeneity in red blood cell (RBC) size, which is routinely reported in complete blood count. RDW is calculated by dividing the standard deviation of the mean cell size by the mean cell volume (MCV) of RBC (5, 6). Therefore, RDW elevation is mathematically caused by decrease in MCV or increase in RBC size variance (7). Previous literatures revealed that the correlation between RDW elevation and impaired erythropoiesis might be attributed to bone marrow dysfunction and poor nutritional status (8). Recently, the RDW level was reported to be influenced by the rate of RBC turnover, which allows persistence of older and smaller cells in circulation. Thus, delayed RBC clearance would have an impact on RBC size variance (9, 10). All the above-mentioned research suggests that increased RDW be an emerging biomarker of abnormal erythrocyte metabolism and survival, potentially representing oxidative stress, inflammation, and a variety of disorders.

It seems hence rational that this simple parameter may reflect the adverse prognosis in many clinical conditions. There are several studies indicating the prognostic performance of advanced RDW level in coronary artery disease (11, 12), heart failure (13, 14), atrial fibrillation (15, 16), and new-onset stroke (17, 18). Existing reports on the relationship between RDW and CKD are comparatively sparse. While some studies showed that RDW was significantly correlated with markers of kidney damage and adverse outcomes in CKD patients (19, 20), others failed to reveal this relationship (21). Despite accumulating evidence, the majority of studies were retrospective, conducted among patients with advanced CKD and/or converged on clinical hard endpoints or ambiguous kidney outcomes.

Furthermore, according to our review of published literature, there are no studies focused on the association between RDW and rate of estimated glomerular filtration rate (eGFR) decline. From this perspective, we attempted to evaluate whether the RDW level was independently associated with eGFR decline and could serve as a novel biomarker for CKD progression among a prospective cohort of patients with stage 1–4 CKD.

## Materials and Methods

### Cohort Participants

**FIGURE 1:**
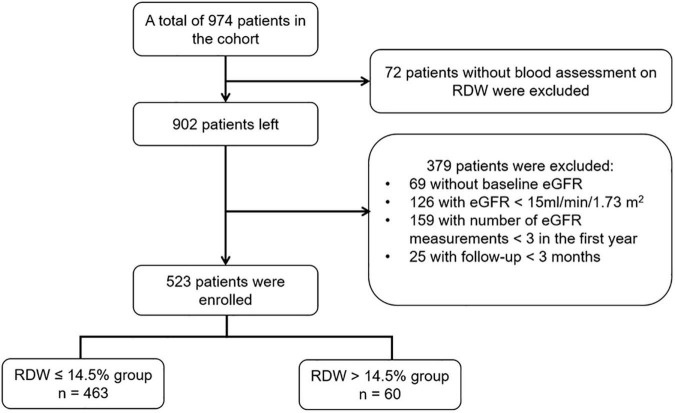
Flow chart of study population. eGFR, estimated glomerular filtration rate; RDW, red blood cell distribution width.

This study was conducted based on the prospective CKD cohort at Peking University First Hospital, which is the first renal division and a renal reference center in China. The patients were from all over China, particularly from northern China. The criteria for the registration consist of aged >18 years, diagnosis of CKD but not on dialysis. Patients were excluded for being with acute kidney injury, having had kidney transplant, active malignancy, or being under pregnancy. The diagnosis of CKD was the presence of persistent anatomical kidney lesions, continuous kidney damage markers or decreased eGFR below 60 ml/min/1.73 m^2^ existing for at least 3 months. All data were collected prospectively. Between January 2003 and December 2020, there were 974 patients with a total of 16,457 records in the database. Among the cohort, 902 patients had at least one measurement of RDW. We identified the date for the first measurement of RDW as baseline date. We excluded patients without eGFR within 3 months prior to the first measurement of RDW (*n* = 69), with a baseline eGFR of < 15 ml/min/1.72 m^2^ (*n* = 126), with number of eGFR measurements <3 over the first-year (*n* = 159), and with follow-up time less than 3 months (*n* = 25), leading to the final study cohort involving 523 participants for the analysis (Figure 1).

Written informed consent was obtained from all participants for use of the clinical data in future studies before they were registered in the cohort. The conduct of the study has been approved by the Ethics Committee of Peking University First Hospital.

### Data Collection

#### Assessment of Red Blood Cell Distribution Width

**FIGURE 2:**
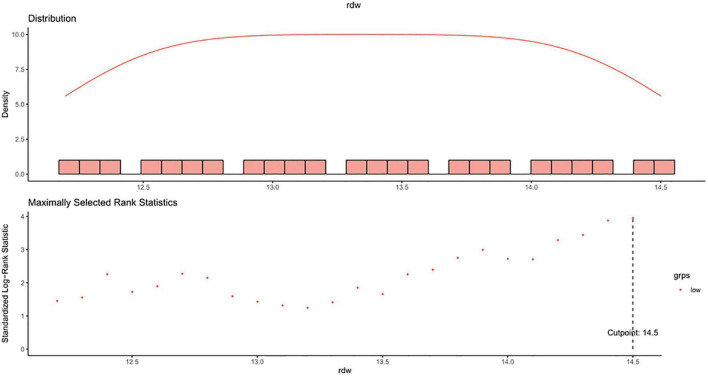
Maximally selected log-rank statistics for cut-off point of RDW. RDW, red blood cell distribution width.

In this study, the optimal cut-off point for RDW as a dichotomous classification of the study participants was chosen based on the maximally selected log-rank statistic for the study outcome, as well as by taking into account the upper limit of normal value of the parameter (14.5%) defined by the local laboratory (the clinical laboratory center of Peking University First Hospital). Coincidentally, the statistic-maximization driven method found out the same threshold of 14.5% (Figure 2).

#### Definition of Hypertension, Anemia, and Diabetes

Hypertension was defined by systolic blood pressure (BP) persistently ≥140 mmHg and/or diastolic BP ≥90 mmHg on two different visits, or self-reported history of hypertension, or use of antihypertensive medications (22). Diabetes was defined by either a fasting blood glucose (FBG) ≥7.0 mmol/L (126 mg/dL) or use of antidiabetic medications (23). We used a criterion for anemia as hemoglobin concentration <13 g/dL in men and <12 g/dL in women, respectively (24).

#### Assessment of Covariates

Patients were followed up regularly every 3–6 months, depending on the patients’ disease condition. We set a time window of 3 months prior to baseline to obtain information of the covariates. Baseline characteristics consisting of demographics (age, sex, and original cause of renal disease), medical history (hypertension and diabetes), medication use and laboratory variables were considered as the most proximate results prior to baseline date. The medication use included iron supplements, erythropoietin (EPO) stimulating agents, angiotensin converting–enzyme inhibitors (ACEI), angiotensin II–receptor blockers (ARB), alpha-blockers, beta-blockers, calcium-channel blockers, and loop diuretics. The laboratory variables included white blood cell (WBC), RBC, hemoglobin, percentage of lymphocyte, MCV, blood glucose, serum albumin, uric acid, serum bicarbonate, calcium, phosphorus, serum iron, urinary albumin-creatinine ratio (UACR), 24-h urine protein, low-density lipoprotein cholesterol (LDL-C), total cholesterol (TCHO), and triglyceride (TG). eGFR was computed using the Chronic Kidney Disease Epidemiology Collaboration (CKD-EPI) equation. The baseline eGFR for each individual established the staging of CKD according to the standards proposed by Kidney Disease: Improving Global Outcomes (KDIGO) guidelines (25). Follow-up data were obtained from the clinical records at the outpatient visits. All of the measurements were performed according to the standard procedure in the clinical laboratory center of Peking University First Hospital.

### Outcomes

To reflect CKD progression, the primary outcome of the study was the slope of eGFR for each individual estimated by linear mixed effect model. We identified a rapid renal function decline as > 5 ml/min/1.73 m^2^/year decline in eGFR, consistent with the 2012 KDIGO guideline (25).

The composite kidney outcome was defined as a confirmed doubling of serum creatinine concentration, a 30% decline in baseline eGFR or incident eGFR <15 ml/min/1.73 m^2^, whichever occurred first. The follow-up was censored at the earliest date among the study end date (31 December 2020), the date of mortality, and the last follow-up date of serum creatinine.

### Statistical Analysis

Descriptive data were presented as number (percentage), mean ± standard deviation (SD) and median with interquartile range, as appropriate. Variables with skewed distributions were log transformed or square root transformed before analysis. Comparisons of between-group variance in baseline characteristics were analyzed using two-sample *t*-test or Mann–Whitney U test for continuous data, as appropriate, and Chi-squared test for categorical data.

Spearman’s correlation analysis was performed to evaluate correlation between RDW value and other laboratory parameters, and correlation coefficients were calculated to determine the strength of these associations. Multivariable analysis was performed among those significant parameters in univariate analysis.

After adjusting for baseline eGFR, we calculated eGFR slope over the entire follow-up period for each patient by using a linear mixed effects model with a random patient-specific intercept and time effect. Category of RDW, follow-up time, category of RDW × time and eGFR recorded at baseline was treated as fixed effect items. An unstructured variance-covariance matrix was employed for each individual. Individual-level eGFR slope was calculated using the fixed-portion linear predictor plus the corresponding predictor in random effects. The distribution was depicted by RDW groups and summarized into categories. The between-group difference in eGFR slope was evaluated by calculating the interaction of RDW groups with time for eGFR decline. Subsequently, univariate and multivariable logistic regression analysis was performed with rapid eGFR decline treated as the outcome. The stepwise elimination with a threshold of *P* < 0.05 was used to select covariates in the multivariable analysis to determine the impact of elevated RDW value on the event.

The incidence rates of composite kidney outcomes were calculated as number of events per 100 person-years. Survival analysis was performed by Kaplan–Meier curves to compare the event rates between RDW categories, with the differences tested by log-rank test. Furthermore, we performed univariate and multivariable Cox proportional hazards regression to estimate the effect of increased RDW value on the composite outcome. Similarly, a stepwise variable selection was employed to select covariates. Hazard ratios were calculated to indicate the strength of association. We checked the proportional hazards assumption by involving an interaction term between the category of RDW and follow-up time to see if there is a statistical significance.

We performed the following sensitivity analyses to test the robustness of our findings. First, we reimplemented our primary analyses using the data over the first-year after enrollment in the cohort. Second, as proposed by several studies, we used an alternative cutoff of 3 ml/min/1.73 m^2^/year to define a rapid renal function decline. Third, given the arbitrary, although not unreasonable, definition of rapid renal function decline, we redefined it as the lowest quartile of eGFR slope among participants included in the cohort. Fourth, to assess whether differences in the frequency of eGFR measurements influence study results, we repeated the primary analyses among patients with at least 2 eGFR measurements during the first-year. Regarding the potential relationship between anemia and RDW value, we tested their cross-product for each of the study outcomes. All analyses were conducted using SAS 9.4 (version 9.4, SAS institute, CA, United States). The threshold for significance was set at *P* < 0.05 (two-side).

## Results

### Baseline Characteristics

In total, 523 patients with a median age 57 (IQR: 44, 69) years were enrolled. Of these patients, 52.77% were men and 38.24% exhibited anemia. The median eGFR at baseline was 37.30 (IQR: 26.86, 49.01) ml/min/1.73 m^2^, and the majority of patients (89.29%) had CKD stage 3 or 4. The cutoff point of RDW level in this study was defined as 14.5%. Thus, enrolled patients were divided into RDW ≤14.5% group and RDW >14.5% group. Baseline characteristics according to dichotomy of RDW are displayed in Table 1. Patients with elevated RDW presented a higher prevalence of anemia and were more likely of receiving iron supplements, EPO stimulating agents, beta-blockers and calcium-channel blockers. In addition, patients with RDW >14.5% had lower values of eGFR, hemoglobin, RBC, MCV, percentage of lymphocyte, serum albumin, calcium, bicarbonate, TCHO, LDL-C and serum iron, but higher levels of UACR, and 24-h urine protein.

**TABLE 1:** Baseline characteristics of the study populations according to the RDW level.

Characteristics	Total (*n* = 523)	RDW group		*P*-value
		RDW ≤ 14.5% (*n* = 463)	RDW > 14.5% (*n* = 60)	
**Demographics**				
Age (years)	57 (44, 69)	56 (43, 69)	60 (48, 73)	0.12
Sex (*n*%)				0.12
Male	276 (52.77)	250 (54.00)	26 (43.33)	
Female	247 (47.23)	213 (46.00)	34 (56.67)	
Cause of renal disease (*n*%)				0.59
Glomerulonephritis	125 (23.90)	111 (23.97)	14 (23.33)	
Diabetic nephropathy	88 (16.83)	80 (17.28)	8 (13.33)	
Hypertensive renal disease	130 (24.86)	112(24.19)	18 (30.00)	
Tubulointerstitial disease	33 (6.31)	32 (6.91)	1 (1.67)	
Polycystic kidney disease	10 (1.91)	8 (1.73)	2 (3.33)	
Pyelonephritis	3 (0.57)	3 (0.65)	0 (0.00)	
Other	36 (6.88)	30 (6.48)	6 (10.00)	
Unknown	98 (18.74)	87 (18.79)	11 (18.33)	
**Clinical**				
Hypertension (*n*%)	151 (28.87)	132 (28.51)	19 (31.67)	0.61
Diabetes (*n*%)	238 (45.51)	213 (46.00)	25 (41.67)	0.52
Anemia (*n*%)				<0.001
Mild	192 (36.71)	153 (33.05)	39 (65.00)	
Moderate	8 (1.53)	4 (0.86)	4 (6.67)	
CKD stages (*n*%)				<0.001
CKD stage 1	9 (1.72)	9 (1.94)	0 (0.00)	
CKD stage 2	47 (8.99)	44 (9.50)	3 (5.00)	
CKD stage 3	293 (56.02)	270 (58.32)	23 (38.33)	
CKD stage 4	174 (33.27)	140 (30.24)	34 (56.67)	
**Laboratory**				
WBC (× 10^12^/L)	6.40 (5.40, 7.60)	6.33 (5.40, 7.60)	6.41 (5.33, 7.55)	0.96
RBC (× 10^12^/L)	4.19 ± 0.64	4.22 ± 0.63	3.92 ± 0.67	<0.001
Hemoglobin (g/L)	129.6 ± 19.7	131.3 ± 19.4	116.1 ± 17.0	<0.001
MCV (fl)	89.96 ± 6.00	90.23 ± 5.80	87.83 ± 7.06	0.01
RDW (%)	13.2 (12.6, 13.8)	13.1 (12.6, 13.6)	15.1 (14.8, 15.7)	<0.001
Lymphocyte (%)	27.39 ± 8.06	27.77 ± 7.88	24.44 ± 8.79	0.002
Albumin (g/dL)	43.0 (40.8, 45.1)	43.1 (41.0, 45.2)	41.9 (38.1, 43.6)	<0.001
Blood glucose (mmol/L)	5.83 ± 1.36	5.80 ± 1.33	6.06 ± 1.54	0.16
Bicarbonate (mmol/L)	24.60 ± 2.93	24.75 ± 2.91	23.43 ± 2.78	0.001
Calcium (mmol/L)	2.31 (2.24, 2.38)	2.32 (2.24, 2.38)	2.26 (2.18, 2.34)	<0.001
Phosphorus (mmol/L)	1.18 (1.04, 1.31)	1.17 (1.03, 1.31)	1.23 (1.10, 1.32)	0.17
Serum iron (μmol/L)	14.99 (13.05, 16.80)	15.18 (13.20, 16.90)	13.41 (11.15, 15.61)	0.002
eGFR (ml/min/1.73 m^2^)	37.30 (26.86, 49.01)	38.27 (27.85, 49.57)	28.40 (22.36, 38.93)	<0.001
UACR (mg/g)	324.71 (128.67, 679.79)	311.43 (117.54, 621.77)	525.70 (279.68, 975.51)	0.004
24-h urine protein (g/24 h)	0.74 (0.21, 1.45)	0.72 (0.19, 1.39)	1.04 (0.59, 1.77)	0.02
Uric acid (μmol/L)	415.3 ± 93.9	415.9 ± 94.8	410.5 ± 87.4	0.68
TG (mmol/L)	1.55 (1.11, 2.24)	1.58 (1.11, 2.27)	1.40 (1.04, 1.89)	0.08
TCHO (mmol/L)	4.55 (3.86, 5.31)	4.57 (3.92, 5.38)	4.08 (3.41, 4.84)	0.003
LDL-C (mmol/L)	2.55 (2.01, 3.09)	2.60 (2.07, 3.14)	2.08 (1.71, 2.77)	<0.001
**Treatment**				
Iron supplements (*n*%)	219 (41.87)	176 (38.01)	43 (71.67)	<0.001
EPO-stimulating agents (*n*%)	143 (27.34)	114 (24.62)	29 (48.33)	<0.001
ACEI or ARB (*n*%)	402 (77.91)	355 (77.85)	47 (78.33)	0.93
Alpha-blockers (*n*%)	145 (28.10)	122 (26.75)	23 (38.33)	0.06
Beta-blockers (*n*%)	124 (24.03)	102 (22.37)	22 (36.67)	0.01
Calcium-channel blockers (*n*%)	379 (73.45)	326 (71.49)	53 (88.33)	0.006
Loop diuretics (*n*%)	188 (36.43)	160 (35.09)	28 (46.67)	0.08

*Data are expressed as mean ± SD, median value (interquartile range) or n (%).*

*ACEI, angiotensin converting–enzyme inhibitors; ARB, angiotensin II–receptor blockers; CKD, chronic kidney disease; eGFR, estimated glomerular filtration rate; EPO, erythropoietin; LDL-C, low-density lipoprotein cholesterol; MCV, mean corpuscular volume; RBC, red blood cell; RDW, red blood cell distribution width; SD, standard deviation; TCHO, total cholesterol; TG, triglyceride; UACR, urinary albumin-creatinine ratio; WBC, white blood cell.*

### Laboratory Variables in Relation to Red Blood Cell Distribution Width

As illustrated in Table 2, the spearman correlation coefficients show that seven parameters correlated inversely with RDW, including RBC, hemoglobin, serum albumin, bicarbonate, calcium, serum iron, and eGFR values in the multivariable adjustment analysis. Conversely, we observed a significant positive association between the RDW level and UACR. Hemoglobin appeared to be the strongest factor correlating with RDW (Spearman’s rank test correlation coefficient, −0.154, *P* < 0.001).

**TABLE 2:** Correlations between baseline RDW and laboratory parameters.

Variables	Univariate analysis		Multivariable analysis	
	*r*	*P*-value	*r*	*P*-value
WBC (× 10^12^/L)	–0.027	0.54		
RBC (× 10^12^/L)	–0.174	< 0.001	–0.125	0.004
Hemoglobin (g/L)	–0.258	< 0.001	–0.154	< 0.001
MCV (fl)	–0.073	0.10		
Lymphocyte (%)	–0.132	0.003	–0.041	0.35
Albumin (g/dL)	–0.205	< 0.001	–0.102	0.02
Blood glucose (mmol/L)	0.076	0.08		
Bicarbonate (mmol/L)	–0.124	0.005	–0.119	0.006
Calcium (mmol/L)	–0.141	0.001	–0.119	0.007
Phosphorus (mmol/L)	0.018	0.69		
Serum iron (μmol/L)	–0.192	< 0.001	–0.093	0.03
eGFR (ml/min/1.73 m^2^)	–0.111	0.01	–0.110	0.01
UACR (mg/g)	0.097	0.03	0.101	0.02
24-h urine protein (g/24 h)	0.060	0.17		
Uric acid (μmol/L)	–0.051	0.25		
TG (mmol/L)	–0.096	0.03	–0.023	0.60
TCHO (mmol/L)	–0.104	0.02	–0.033	0.45
LDL-C (mmol/L)	–0.101	0.02	–0.061	0.17

*eGFR, estimated glomerular filtration rate; LDL-C, low-density lipoprotein cholesterol; MCV, mean corpuscular volume; RBC, red blood cell; RDW, red blood cell distribution width; TCHO, total cholesterol; TG, triglyceride; UACR, urinary albumin-creatinine ratio; WBC, white blood cell.*

### Associations of Red Blood Cell Distribution Width With Estimated Glomerular Filtration Rate Decline

The observed change rate in eGFR after a median follow-up of 26 (IQR: 12, 36) months was −1.86 (95% CI: −2.27, −1.45) ml/min/1.73 m^2^/year in the RDW ≤14.5% group compared with −3.48 (95% CI: −4.84, −2.12) ml/min/1.73 m^2^/year in the RDW >14.5% group, with a significant between-group difference of 1.62 (95% CI: 0.20, 3.04) ml/min/1.73 m^2^/year (*P* = 0.03).

**FIGURE 3:**
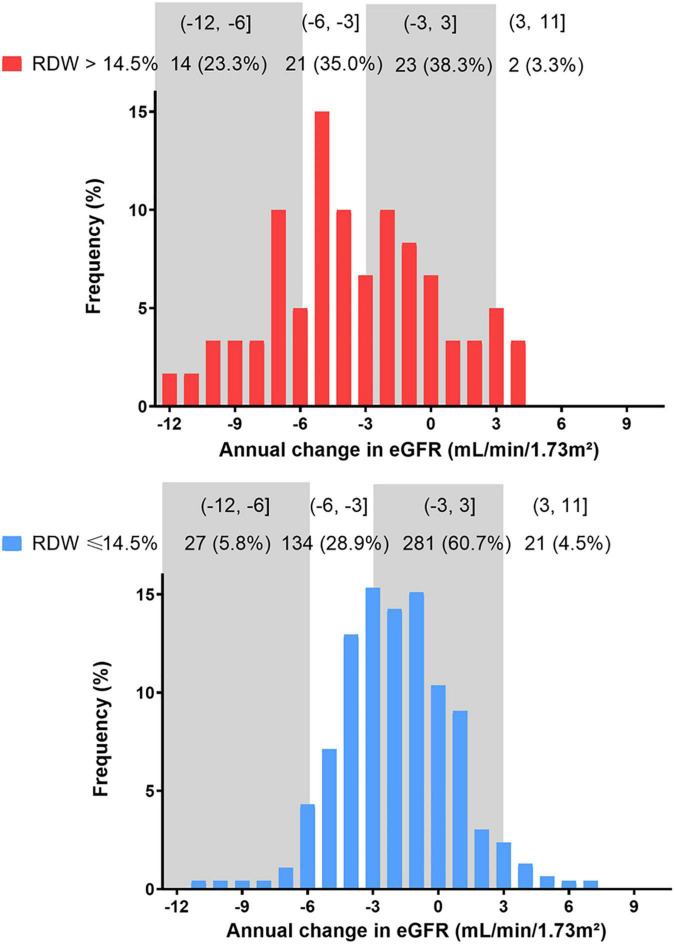
Distribution of eGFR slope during the entire study period by RDW group. eGFR, estimated glomerular filtration rate.

The distribution of eGFR slope was graphically depicted across RDW groups in Figure 3. In total, 29.64 and 7.84% of enrolled patients suffered an eGFR decline rate of 3–6 ml/min/1.73 m^2^/year and over 6 ml/min/1.73 m^2^/year, with higher proportions in those with RDW >14.5% (Figure 3).

Consequently, a total of 65 patients (12.43%) underwent rapid renal function decline (eGFR loss >5 ml/min/1.73 m^2^/year). 44 (9.50%) of these were in the RDW ≤14.5% group and 21 (35.00%) in the RDW >14.5% group (Table 3).

**TABLE 3:** Univariate and multivariable adjusted odds ratios for rapid eGFR decline.

Variables	Events of RFD (*n*, %)	Univariate analysis		Multivariable analysis*^a^*	
		OR (95% CI) for RFD	*P*-value	OR (95% CI) for RFD	*P*-value
RDW ≤ 14.5%	44 (9.50)	Ref		Ref	
RDW > 14.5%	21 (35.00)	5.13 (2.77, 9.48)	<0.001	6.79 (3.08, 14.97)	<0.001

*Rapid function decline was defined as eGFR loss >5 ml/min/1.73 m^2^/year.*

*^a^The model was further adjusted for tubulointerstitial disease as the primary cause of renal failure, usage of iron supplements (yes vs. no), usage of EPO-stimulating agents (yes vs. no), usage of loop diuretics (yes vs. no), usage of alpha-blockers (yes vs. no), usage of calcium-channel blockers (yes vs. no), log (10)-transformed age, percentage of lymphocyte, natural log-transformed baseline eGFR, log (10)-transformed albumin, log (10)-transformed calcium, natural log-transformed UACR, log (10)-transformed 24-h urine protein, and log (10)-transformed LDL-C.*

*ACEI, angiotensin converting–enzyme inhibitors; ARB, angiotensin II–receptor blockers; CI, confidence interval; eGFR, estimated glomerular filtration rate; EPO, erythropoietin; LDL-C, low-density lipoprotein cholesterol; RDW, red blood cell distribution width; RFD, rapid function decline; OR, odds ratio; UACR, urinary albumin-creatinine ratio.*

Multivariable logistic regression reveals that elevated RDW values at baseline was associated with rapid eGFR deterioration. Adjusted odds ratio exceeded 5.79 for RDW >14.5% with 95% confidence interval of 3.08–14.97 (*P* < 0.001) (Table 3). Covariate selection is displayed in Supplementary Table 1.

### Associations of Red Blood Cell Distribution Width With Composite Kidney Outcomes

**FIGURE 4:**
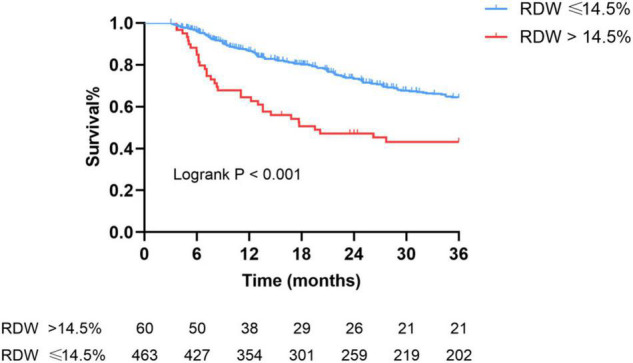
Kaplan–Meier survival curves of composite kidney outcomes according to RDW levels. (Log-rank test, *P* < 0.001). The numbers below the *x*-axis indicate the number of event-free patients observed at 6, 12, 18, 24, 30, and 36 months.

There were 172 events (32.89%) of composite kidney outcomes including 33 (55.00%) in the RDW >14.5% group and 139 (30.02%) in the RDW ≤14.5% group, following crude incidence rates of 32.3 and 14.7 per 100 patient-years, respectively. The overall kidney survival rate was 64.20% in the RDW ≤14.5% group and 43.20% in the RDW >14.5% group (*P* for log-rank test < 0.001) (Figure 4).

Table 4 shows the multivariable adjusted hazard ratios of RDW categories for progression to composite kidney outcomes. Presence of elevated RDW was significantly associated with kidney events (fully adjusted hazard ratio: 1.51, 95% CI: 1.02–2.23, *P* = 0.03). Nonetheless, interactions between the concentration of hemoglobin and baseline eGFR were not significant through all models (data not shown).

**TABLE 4:** Hazard ratios (95% CI) for composite kidney outcomes over the entire study period.

Variables	RDW ≤ 14.5% (*n* = 463)	RDW > 14.5% (*n* = 60)	*P*-value
**Entire study period analysis**			
Events (*n*, %)			< 0.001
Doubling of SCR	5 (1.08)	0	
30% decline in eGFR	116 (25.05)	28 (46.67)	
eGFR <15 ml/min/1.73 m^2^	56 (12.10)	14 (23.33)	
**HR (95% CI) for composite kidney outcomes**			
Model 1	Ref	2.24 (1.53, 3.28)	< 0.001
Model 2	Ref	1.55 (1.05, 2.83)	0.02
Model 3	Ref	1.51 (1.02, 2.23)	0.03

*Model 1: non-adjusted.*

*Model 2: adjusted for sex, log (10)-transformed age, history of hypertension (yes vs. no), usage of iron supplements (yes vs. no), usage of EPO-stimulating agents (yes vs. no), usage of ACEI or ARB (yes vs. no), usage of beta-blockers (yes vs. no), usage of alpha-blockers (yes vs. no), usage of calcium-channel blockers (yes vs. no).*

*Model 3: adjusted for Model 2 + RBC, hemoglobin, percentage of lymphocyte, MCV, log (10)-transformed serum iron, natural log-transformed baseline eGFR, natural log-transformed UACR, log (10)-transformed 24-h urine protein, log (10)-transformed albumin, bicarbonate, log (10)-transformed calcium, log (10)-transformed phosphorus, uric acid, blood glucose, and log (10)-transformed LDL-C.*

*ACEI, angiotensin converting–enzyme inhibitors; ARB, angiotensin II–receptor blockers; CI, confidence interval; eGFR, estimated glomerular filtration rate; EPO, erythropoietin; HR, hazard ratio; LDL-C, low-density lipoprotein cholesterol; MCV, mean corpuscular volume; RDW, red blood cell distribution width; RBC, red blood cell; UACR, urinary albumin-creatinine ratio.*

### Sensitivity Analyses

Similar associations were confirmed in several sensitivity analyses. Individual 1-year eGFR slope consistently diverged significantly between RDW >14.5% group (−5.29 [95% CI: −7.97, −2.62] ml/min/1.73 m^2^/year) and RDW ≤14.5% group (−1.60 [95% CI: −2.42, −0.79] ml/min/1.73 m^2^/year) with between-group difference of 3.69 (95% CI: 0.89, 6.49) ml/min/1.73 m^2^/year (*P* = 0.009). (Supplementary Table 2 and Supplementary Figure 1). The association of RDW with rapid function decline and composite kidney outcomes remained consistently significant over the first-year after enrollment (Supplementary Tables 2, 3 and Supplementary Figure 2). Consistent findings were observed when rapid function decline was redefined by the lowest quartile of eGFR slope or an alternative cutoff of 3 ml/min/1.73 m^2^/year (Supplementary Tables 4, 5). Among patients with at least 2 eGFR measurements available over the first-year, we found that RDW >14.5% remained a statistically significant association with fast eGFR decline (Supplementary Table 6).

## Discussion

In this prospective study of 523 patients with non-dialysis-dependent CKD (NDD-CKD) stage 1–4, we demonstrated that RDW >14.5% was independently associated with faster eGFR loss rate and the presence of rapid kidney disfunction. Survival analysis provided further validation of observed associations between RDW and adverse CKD prognostic outcomes.

Despite multiple epidemiological studies, the impact of RDW on the prognosis of kidney disease remains controversial and data observed in populations with earlier stages of CKD are limited. To date, higher RDW level has been proved to associate with all-cause mortality risk in both hemodialysis (26) and peritoneal dialysis patients (27). A retrospective study on 282 NDD-CKD patients found that RDW ≥14.5% was associated with an enhanced risk of major composite cardiovascular outcomes (28). Additionally, a significantly higher risk of requiring dialysis or doubling of serum creatinine was observed in NDD-CKD patients with sustained, higher RDW (29). On the contrary, a large registry-based cohort study did not confirm that RDW was a prognostic factor of persistent requirement for dialysis therapy (21). Given the unclear relationship between RDW and CKD prognosis, our study extended the previous results by demonstrating that RDW was correlated with rapid renal function loss, concerning both long-term effects and short-term effects over 1-year follow-up.

Since some inherent explanations of RDW variation have been previously studied, including aging, inflammation, metabolic disorders and nutritional deficiencies (30), we investigated the associations between RDW and possibly related variables. Notably significant correlations between RDW value and a series of laboratory variables including erythrocytes count, hemoglobin, initial eGFR, UACR, serum albumin, and serum iron were observed in this study, indicating the potential role of RDW as manifestation of malnutrition and impaired erythropoiesis, thus poorer overall health, which was consistent with previous articles (5). Although it is hypothesized that factors like age, sex, lipid profiles, co-existing diabetes, or hypertension might also associate with RDW, we found no constant connection between RDW level and these variables after adjustments for covariates.

Previous studies reported that RDW is either normal or elevated. The circumstance of a RDW value below the normal reference range is infrequent and considered to be clinically meaningless (31, 32). Taken together, we noted that elevated RDW was associated with higher risk of CKD progression, after adjusting for baseline eGFR, albuminuria, serum iron, blood level of hemoglobin, and albumin. Although the hidden mechanisms were not fully understood, we have several speculations. Very importantly, the presence of anemia was associated with adverse clinical outcomes in CKD patients (33). It was demonstrated in experimental researches that RDW first became abnormal than other measures including hemoglobin, MCV and transferrin saturation as iron deficiency anemia (IDA) developed (34), suggesting an early biochemical clue. Because advanced CKD often results in deficiency of EPO production and impaired iron absorption, RDW may be an essential reflection of progressive renal disease. Another reasonable explanation is probably related to systemic inflammation. A strong, graded association between RDW and C-reactive protein (CRP), along with erythrocyte sedimentation rate (ESR) independently of multiple confounders was revealed in a large-size cohort (35). Chronic inflammation and subsequent oxidative stress were suspected to serve as the key intermediates linking increased RDW value to kidney injury (36). However, the pathophysiologic effect of inflammatory biomarkers could not be assessed in our study. In addition, whether RDW is simply an epiphenomenon of underlying inflammation as the result of kidney disease or an essential risk factor of progressive renal damage, remained to be confirmed in future exploration. On top of that, atherosclerosis is commonplace in the context of CKD, and may help explain the correlates of RDW and cardiovascular risk (37). Previous studies concluded that the cholesterol content of erythrocytes membranes was positively associated with RDW values independently of inflammatory, nutritional and hematological confounders (38). Partially owing to the fact that anisocytosis might result in turbulence of blood flow, RDW was associated with thrombotic occlusions and vascular injuries in glomeruli (39). In summary, impaired body metabolism might contribute to elevated RDW level and thereby facilitate inflammatory process and impaired antioxidant status, thus triggering adverse kidney outcomes.

The strengths of this study included the sequential measurements of serum creatinine and calculation of eGFR slope. As former studies primarily concentrated on clinical hard endpoints, this is the first research that evaluated the impact of RDW on eGFR decline rate, a dynamic parameter with strong prognostic utility (40), in NDD-CKD patients. Previous studies have emphasized that the accuracy in estimating eGFR slope lies in the number of repeated eGFR measurements (41). Our study cohort was conducted in a busy outpatient nephrology clinic, providing frequent measurements of eGFR, which helped mitigate bias from acute effect of intervention or random fluctuations of eGFR. Also, we used linear mixed effects models to calculate eGFR slope, which are more robust than ordinary linear regression models when the baseline eGFR values significantly vary among study participants. As there is no consensus definition of a rapid eGFR decline, our primary analysis was performed using the original criteria recommended by the KDIGO guideline (eGFR decline >5 ml/min/1.73 m^2^/year) (25). Furthermore, as proposed by other studies (42), we conducted the sensitivity analysis under alternative thresholds of eGFR decline >3 ml/min/1.73 m^2^/year and that based on a natural distribution of eGFR change in our own study sample. Since the length of observation may influence the effect of the exposure, another sensitivity analysis was carried out using only the first-year data. Additionally, to mitigate the possible selection bias, we added participants with only 2 eGFR available during the first-year observation period into analysis. Overall, the above sensitivity analyses suggested consistent results.

There are several limitations that must be acknowledged. Firstly, this cohort with an observational design was conducted at a single center on a moderate sample size, which might restrict the cause-and-effect relationship and the extrapolation of findings to other populations. Secondly, the 3-year follow-up was relatively short and thus confined implication for long-term effects on CVD events or death. Thirdly, our database is lacking inflammatory biomarkers, such as CRP or interleukin-6 (IL-6), because only a small proportion of patients maintained routine tests of these indices. Fourthly, our data source was also short of information on nutrition intake or anthropometric parameters. Hence, serum albumin, TG, TC, and LDL-C were regarded as substitute nutritional data. Finally, some unknown confounders might affect the observed associations even after extensive adjustments.

## Conclusion

In conclusion, our results revealed that RDW >14.5% was independently associated with rapid CKD progression and adverse kidney outcomes, after adjusting for initial kidney function, nutritional state, and presence of anemia. Since chronic inflammation or ineffective erythropoiesis might be prevalent silently until a later stage in the course of CKD progression, RDW might serve as an indicator for the disease progression. With its wide availability and low cost for measurement, RDW might be useful in monitoring NDD-CKD patients.

## Data Availability Statement

The datasets generated and analyzed during the current study are available from the corresponding authors on reasonable request.

## Ethics Statement

The studies involving human participants were reviewed and approved by the Ethics Committee of Peking University First Hospital. The patients/participants provided their written informed consent to participate in this study.

## Author Contributions

XD searched the literature, designed the study, analyzed the data, interpreted the results, and drafted the manuscript. LZ and JW supervised the study and revised the manuscript. M-HZ, LZ, FW, BG, and JW were involved in data collection and data cleaning. All authors have read and approved the final manuscript.

## Conflict of Interest

The authors declare that the research was conducted in the absence of any commercial or financial relationships that could be construed as a potential conflict of interest.

## Publisher’s Note

All claims expressed in this article are solely those of the authors and do not necessarily represent those of their affiliated organizations, or those of the publisher, the editors and the reviewers. Any product that may be evaluated in this article, or claim that may be made by its manufacturer, is not guaranteed or endorsed by the publisher.
