# Supplementary material for: Red Blood Cell Distribution Width Is Associated With Adverse Kidney Outcomes in Patients With Chronic Kidney Disease
Source: Front Med (Lausanne). 2022 Jun 9;9:877220. doi: 10.3389/fmed.2022.877220 (PMC9218182; doi:10.3389/fmed.2022.877220)
Supplement: Supplementary file 1 [file Data_Sheet_1.docx]

Supplementary Material

# Supplementary Table 1. Univariate and multivariable adjusted odds ratios for rapid eGFR decline.

| **Variables** | **Univariate analysis** | | **Multivariable analysis** | |
| --- | --- | --- | --- | --- |
|  | **OR (95% CI)** **for RFD** | ***P* value** | **OR (95% CI) for RFD** | ***P* value** |
| RDW ≤ 14.5% | ref |  | ref |  |
| RDW > 14.5% | 5.13 (2.77, 9.48) | < 0.001 | 6.79 (3.08, 14.97) | < 0.001 |
| Sex (Male) | 1.13 (0.67, 1.90) | 0.65 |  |  |
| Tubulointerstitial disease (Yes) | 2.82 (1.14, 6.99) | 0.02 | 1.47 (0.43, 3.49) | 0.12 |
| Hypertension (Yes) | 1.31 (0.75, 2.27) | 0.35 |  |  |
| Diabetes (Yes) | 1.23 (0.53, 1.51) | 0.67 |  |  |
| iron supplements (Yes) | 2.00 (1.19, 3.39) | < 0.001 | 2.51 (1.18, 5.35) | 0.02 |
| EPO-stimulating agents (Yes) | 1.80 (1.05, 3.10) | 0.03 | 0.85 (0.34, 2.14) | 0.73 |
| ACEI or ARB (Yes) | 0.93 (0.61, 1.44) | 0.76 |  |  |
| Alpha-blockers (Yes) | 0.40 (0.27, 0.59) | < 0.001 | 1.60 (0.76, 3.35) | 0.21 |
| Beta-blockers (Yes) | 0.67 (0.45, 1.02) | 0.06 |  |  |
| Calcium-channel blockers (Yes) | 0.62 (0.40, 0.94) | 0.02 | 1.14 (0.48, 2.68) | 0.76 |
| Loop diuretics (Yes) | 0.59 (0.41, 0.85) | 0.005 | 1.57 (0.83, 2.98) | 0.16 |
| Log-age per SD | 0.11 (0.02, 0.66) | 0.02 | 0.29 (0.03, 3.15) | 0.31 |
| Log-WBC per SD | 1.02 (0.11, 9.39) | 0.23 |  |  |
| RBC (×10^12^/L) per unit | 1.01 (0.67, 1.52) | 0.96 |  |  |
| Hemoglobin (g/L) per unit | 0.99 (0.98, 1.01) | 0.23 |  |  |
| MCV (fl) per unit | 0.96 (0.91, 1.01) | 0.10 |  |  |
| Lymphocyte (%) per unit | 0.95 (0.92, 0.99) | 0.01 | 0.98 (0.94, 1.03) | 0.54 |
| Log-Albumin per SD | 0.91 (0.87, 0.95) | < 0.001 | 0.92 (0.88, 0.97) | 0.002 |
| Blood glucose (mmol/L) per unit | 1.16 (0.98, 1.34) | 0.08 |  |  |
| Bicarbonate (mmol/L) per unit | 1.05 (0.96, 1.15) | 0.31 |  |  |
| Log-Calcium per SD | 0.10 (0.02, 0.55) | 0.004 | 0.02 (0.00, 4.32) | 0.80 |
| Log-Phosphorus per SD | 1.45 (0.05, 9.77) | 0.83 |  |  |
| Log-Serum iron per SD | 1.82 (0.95, 3.50) | 0.07 |  |  |
| Log-eGFR per SD | 3.00 (1.59, 5.67) | < 0.001 | 19.05 (7,50, 48.40) | < 0.001 |
| Log-UACR per SD | 1.61 (1.29, 2.00) | < 0.001 | 1.13 (0.78, 1.63) | 0.53 |
| Log-24-hour urine protein per SD | 1.70 (1.35, 2,14) | < 0.001 | 1.49 (1.39, 2.48) | < 0.001 |
| Uric acid (µmol/L) per unit | 0.99 (0.98, 1.03) | 0.52 |  |  |
| Log-TG per SD | 1.11 (0.32, 3.83) | 0.87 |  |  |
| Log-TCHO per SD | 0.46 (0.11, 4.97) | 0.51 |  |  |
| Log-LDL-C per SD | 0.25 (0.08, 0.72) | 0.01 | 0.27 (0.08, 0.95) | 0.04 |

**Note:** rapid function decline was defined as eGFR loss > 5 ml/min/1.73m^2^/year.

**Abbreviations:** ACEI: angiotensin converting–enzyme inhibitors, ARB: angiotensin II–receptor blockers, CI: confidence interval, eGFR: estimated glomerular filtration rate, EPO: erythropoietin, LDL-C: low-density lipoprotein cholesterol, MCV: mean corpuscular volume, RBC: red blood cell, RDW: red blood cell distribution width, SD: Standard deviation, TCHO: total cholesterol, TG: triglyceride, UACR: urinary albumin-creatinine ratio, WBC: white blood cell.

# Supplementary Table 2. Univariate and multivariable adjusted odds ratios for rapid eGFR decline over the first year after enrollment.

| **Variables** | **eGFR slope** | **Events of RFD (n, %)** | **Univariate analysis** | | **Multivariable analysis ^a^** | |
| --- | --- | --- | --- | --- | --- | --- |
|  | **(mL/min/1.73m^2^/year)** |  | **OR (95% CI) for RFD** | ***P* value** | **OR (95% CI) for RFD** | ***P* value** |
| ***One Year analysis*** | | |  |  |  |  |
| **RDW ≤ 14.5%** | -1.60 (-2.42, -0.79) | 124 (26.78) | ref |  | ref |  |
| **RDW > 14.5%** | -5.29 (-7.97, -2.62) | 27 (45.00) | 2.24 (1.29, 3.87) | 0.004 | 2.11 (1.15, 3.86) | 0.02 |

**Note:** rapid function decline was defined as eGFR loss > 5 ml/min/1.73m^2^/year. ^a^ The model was further adjusted for sex (male vs. female), usage of iron supplements (yes vs. no), usage of EPO-stimulating agents (yes vs. no), usage of beta-blockers (yes vs. no), usage of alpha-blockers (yes vs. no), usage of calcium-channel blockers (yes vs. no), usage of ACEI or ARB (yes vs. no), log (10)-transformed age, percentage of lymphocyte, log (10)-transformed albumin, log (10)-transformed calcium, natural log-transformed baseline eGFR, natural log-transformed UACR and 24-hour urine protein.

**Abbreviations:** ACEI: angiotensin converting–enzyme inhibitors, ARB: angiotensin II–receptor blockers, CI: confidence interval, eGFR: estimated glomerular filtration rate, EPO: erythropoietin, RDW: red blood cell distribution width, RFD: rapid function decline, OR: odds ratio, UACR: urinary albumin-creatinine ratio.

# Supplementary Table 3. Hazard ratios (95% CI) for composite kidney outcomes over the first year after enrollment.

|  | **Variables** | **RDW ≤ 14.5%**  **(n = 463)** | **RDW > 14.5%**  **(n = 60)** | ***P* value** |
| --- | --- | --- | --- | --- |
|  | ***One Year analysis*** | |  |  |
| **Events (n, %)** | |  |  | < 0.001 |
|  | **doubling of SCR** | 2 (0.43) | 0 |  |
|  | **30% decline in eGFR** | 40 (8.64) | 17 (28.33) |  |
|  | **eGFR<15 ml/min/1.73m^2^** | 33 (7.13) | 8 (13.33) |  |
| **HR (95% CI) for composite kidney outcomes** | | |  |  |
|  | **Model 1** | ref | 3.19 (1.93, 5.25) | < 0.001 |
|  | **Model 2** | ref | 1.84 (1.11, 3.07) | 0.02 |
|  | **Model 3** | ref | 1.75 (1.05, 3.00) | 0.04 |

Model 1: non-adjusted.

Model 2: adjusted for sex, log (10)-transformed age, history of hypertension (yes vs. no), usage of iron supplements (yes vs. no), usage of EPO-stimulating agents (yes vs. no), usage of ACEI or ARB (yes vs. no), usage of beta-blockers (yes vs. no), usage of alpha-blockers (yes vs. no), usage of calcium-channel blockers (yes vs. no).

Model 3: adjusted for Model 2 + RBC, hemoglobin, MCV, log (10)-transformed serum iron, natural log-transformed baseline eGFR, natural log-transformed UACR, log (10)-transformed 24-hour urine protein, log (10)-transformed albumin, bicarbonate, log (10)-transformed calcium, log (10)-transformed phosphorus, uric acid and blood glucose.

**Abbreviations:** ACEI: angiotensin converting–enzyme inhibitors, ARB: angiotensin II–receptor blockers, CI: confidence interval, eGFR: estimated glomerular filtration rate, EPO: Erythropoietin, HR: hazard ratio, MCV: mean corpuscular volume, RBC: red blood cell, RDW: red blood cell distribution width, UACR: urinary albumin-creatinine ratio.

# Supplementary Table 4. Univariate and multivariable adjusted odds ratios for redefined rapid function decline.

| **Variables** | **Events of RFD (n, %)** | **Univariate analysis** | | **Multivariable analysis** ^a^ | |
| --- | --- | --- | --- | --- | --- |
|  |  | **OR (95% CI) for RFD** | ***P* value** | **OR (95% CI) for RFD** | ***P* value** |
| **RDW ≤ 14.5%** | 161 (34.77) | ref |  | ref |  |
| **RDW > 14.5%** | 35 (58.33) | 2.63 (1.52, 4.54) | < 0.001 | 2.22 (1.24, 4.00) | 0.008 |

**Note:** rapid function decline was redefined as eGFR loss > 3 ml/min/1.73m^2^/year. ^a^ The model was further adjusted for tubulointerstitial disease as the primary cause of renal failure, usage of iron supplements (yes vs. no), usage of EPO-stimulating agents (yes vs. no), usage of ACEI or ARB (yes vs. no), usage of loop diuretics (yes vs. no), usage of alpha-blockers (yes vs. no), usage of calcium-channel blockers (yes vs. no), log (10)-transformed age, percentage of lymphocyte, natural log-transformed baseline eGFR, log (10)-transformed albumin, log (10)-transformed calcium, log (10)-transformed phosphorus, natural log-transformed UACR, log (10)-transformed 24-hour urine protein and log (10)-transformed LDL-C.

**Abbreviations:** ACEI: angiotensin converting–enzyme inhibitors, ARB: angiotensin II–receptor blockers, CI: confidence interval, eGFR: estimated glomerular filtration rate, EPO: erythropoietin, LDL-C: low-density lipoprotein cholesterol, RDW: red blood cell distribution width, RFD: rapid function decline, OR: odds ratio, UACR: urinary albumin-creatinine ratio.

# Supplementary Table 5. Quartiles of eGFR slope and odds ratios for redefined rapid function decline.

| **Variables** | **eGFR slope distribution (mL/min/1.73m^2^/ year)** | | | | | | **Univariate analysis** | | **Multivariable analysis** | |
| --- | --- | --- | --- | --- | --- | --- | --- | --- | --- | --- |
| **(n, %)** | **≤ q1** | | | **(q1, q2］** | **(q2, q3］** | **> q3** | **OR (95% CI) for RFD^*^** | ***P* value** | **OR (95% CI) for RFD^*^** | ***P* value** |
| ***Entire study period analysis*** | | **≤ -4.0** | **(-4.0, -2.1］** | | **(-2.1, -0.3］** | **> -0.3** |  |  |  |  |
| **RDW ≤ 14.5%** | 99 (21.38) | | | 125 (27.00) | 121 (26.13) | 118 (25.49) | ref |  | ref |  |
| **RDW > 14.5%** | 30 (50.00) | | | 8 (13.33) | 9 (15.00) | 13 (21.67) | 3.68 (2.12, 6.39) | < 0.001 | 2.88 (1.56, 5.33) ^a^ | 0.002 |
| ***One Year analysis*** | | **≤ -5.9** | **(-5.9, -2.2］** | | **(-2.2, 1.4］** | **> 1.4** |  |  |  |  |
| **RDW ≤ 14.5%** | 103 (22.25) | | | 120 (25.92) | 121 (26.13) | 119 (25.70) | ref |  | ref |  |
| **RDW > 14.5%** | 27 (45.00) | | | 11 (18.33) | 11 (18.33) | 11 (18.33) | 2.86 (1.64, 4.98) | < 0.001 | 2.08 (1.15, 3.78) ^b^ | 0.02 |

**Note:** rapid function decline was redefined as the lowest quartile of eGFR slope. ^a^ The model was further adjusted for sex (male vs. female), usage of iron supplements (yes vs. no), usage of EPO-stimulating agents (yes vs. no), usage of alpha-blockers (yes vs. no), usage of ACEI or ARB (yes vs. no), usage of calcium-channel blockers (yes vs. no), log (10)-transformed age, log (10)-transformed albumin, hemoglobin, MCV, percentage of lymphocyte, log (10)-transformed serum iron, natural log-transformed baseline eGFR, natural log-transformed UACR and 24-hour urine protein, log (10)-transformed LDL-C. ^b^ The model was further adjusted for sex (male vs. female), usage of iron supplements (yes vs. no), usage of EPO-stimulating agents (yes vs. no), usage of alpha-blockers (yes vs. no), usage of ACEI or ARB (yes vs. no), log (10)-transformed age, log (10)-transformed albumin, hemoglobin, MCV, percentage of lymphocyte, natural log-transformed baseline eGFR, natural log-transformed UACR and 24-hour urine protein.

**Abbreviations:** ACEI: angiotensin converting–enzyme inhibitors, ARB: angiotensin II–receptor blockers, CI: confidence interval, eGFR estimated glomerular filtration rate, EPO: erythropoietin, LDL-C: low-density lipoprotein cholesterol, MCV: mean corpuscular volume, RDW: red blood cell distribution width, RFD: rapid function decline, OR: odds ratio, UACR: urinary albumin-creatinine ratio.

# Supplementary Table 6. Univariate and multivariable adjusted odds ratios for rapid eGFR decline among patients with at least 2 eGFR available in the first year.

| **Variables** | **eGFR slope** | **Events of RFD (n, %)** | **Univariate analysis** | | **Multivariable analysis** | |
| --- | --- | --- | --- | --- | --- | --- |
|  | **(mL/min/1.73m^2^/ year)** |  | **OR (95% CI) for RFD** | ***P* value** | **OR (95% CI) for RFD** | ***P* value** |
| ***Entire study period analysis*** | | |  |  |  |  |
| **RDW** **≤ 14.5% (n = 499)** | -1.88 (-2.19, -1.57) | 23 (4.61) | ref |  | ref |  |
| **RDW > 14.5% (n = 64)** | -3.61 (-4.95, -2.26) | 9 (14.06) | 3.39 (1.49, 7.69) | 0.004 | 3.54 (1.45, 8.67) ^a^ | 0.006 |
| ***One Year analysis*** | |  |  |  |  |  |
| **RDW ≤ 14.5% (n = 499)** | -2.15 (-3.00, -1.31) | 148 (29.66) | ref |  | ref |  |
| **RDW > 14.5% (n = 64)** | -10.46 (-17.56, -3.35) | 28 (43.75) | 1.89 (1.08, 3.34) | 0.03 | 2.83 (1.54, 5.20) ^b^ | 0.001 |

**Note:** rapid function decline was defined as eGFR loss > 5 ml/min/1.73m^2^/year. The between-group difference of total eGFR slope is 1.72 (95% CI: 0.34, 3.10) ml/min/1.73m^2^/year (*P* = 0.01). The between-group difference of 1-year eGFR slope is 8.30 (95% CI: 1.15, 15.45) ml/min/1.73m^2^/year (*P* = 0.02). ^a^ The model was further adjusted for sex (male vs. female), usage of iron supplements (yes vs. no), usage of EPO-stimulating agents (yes vs. no), usage of beta-blockers (yes vs. no), usage of alpha-blockers (yes vs. no), usage of calcium-channel blockers (yes vs. no), usage of ACEI or ARB (yes vs. no), log (10)-transformed age, log (10)-transformed albumin, natural log-transformed baseline eGFR, bicarbonate, natural log-transformed UACR and 24-hour urine protein. ^b^ The model was further adjusted for sex (male vs. female), usage of iron supplements (yes vs. no), usage of EPO-stimulating agents (yes vs. no), usage of beta-blockers (yes vs. no), usage of alpha-blockers (yes vs. no), usage of calcium-channel blockers (yes vs. no), usage of ACEI or ARB (yes vs. no), log (10)-transformed age, log (10)-transformed albumin, natural log-transformed baseline eGFR, log (10)-transformed calcium, natural log-transformed UACR and 24-hour urine protein.

**Abbreviations:** ACEI: angiotensin converting–enzyme inhibitors, ARB: angiotensin II–receptor blockers, CI: confidence interval, eGFR: estimated glomerular filtration rate, EPO: erythropoietin, RDW: red blood cell distribution width, RFD: rapid function decline, OR: odds ratio, UACR: urinary albumin-creatinine ratio.


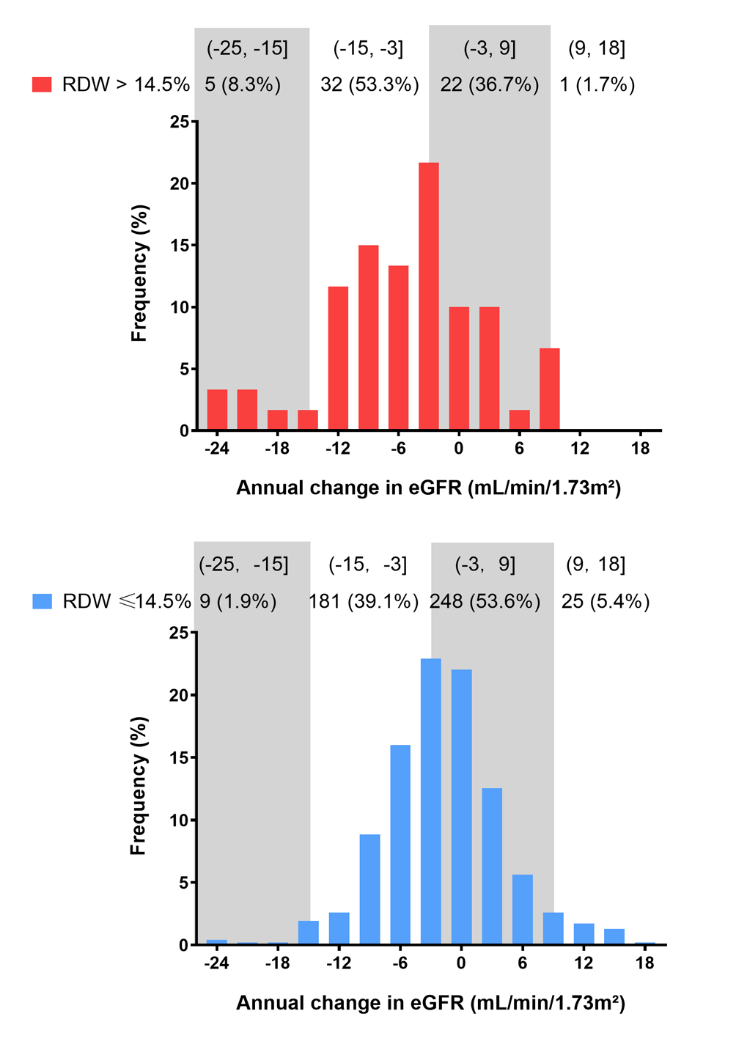


# Supplementary Figure 1. Distribution of eGFR slope over the first year after enrollment by RDW group.

# Abbreviations: eGFR: estimated glomerular filtration rate.


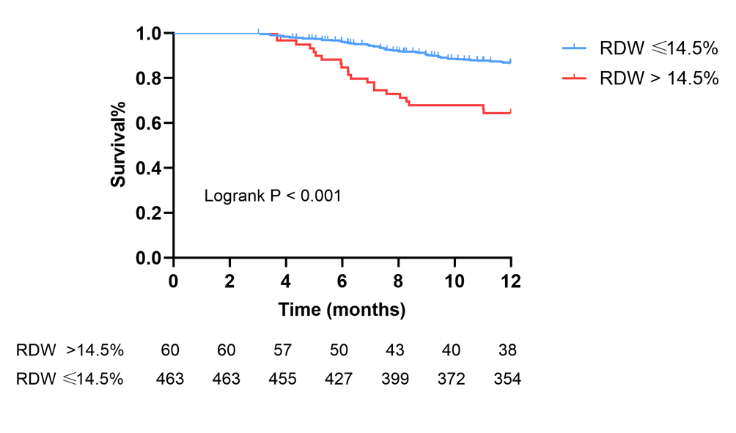


# Supplementary Figure 2. Kaplan-Meier survival curves of composite kidney outcomes over the first year after enrollment according to RDW levels. (Log-rank test, P < 0.001). The numbers below the x-axis indicate the number of event-free patients observed at 2, 4, 6, 8, 10 and 12 months.
